# Scopolamine and Morphine in Labor—A Clinical Study of Twenty-One Cases

**Published:** 1915-10

**Authors:** J. L. Campbell

**Affiliations:** Atlanta, Ga.


### SCOPOLAMINE AND MORPHINE IN LABOR—A CLINICAL STUDY OF TWENTY-ONE CASES.

J. L. Campbell, M. D., F. A. C. S., Atlanta, Ga.

In November, 1913, Professor Kronenig visited this country and read a paper before the American Congress of Surgeons and the Chicago Gynecological Society entitled (1) "The Difference Between the Older and Newer Treatment by X-Ray and Radium in Gynecological Diseases." The greater part of this paper was devoted to a discussion of the plan then in use in the Freiberg Clinic for producing painless labor by the administration of Scopolamine and Morphine.

In June of the next year McClure's Magazine published an article on the subject which excited public interest in the belief, that the dread period of child-birth could be rendered painless, or so nearly so, that the mother on awaking would have no recollection of the ordeal through which she had passed. In consequence of this, and other articles in the popular magazines, nearly every women expecting to become a mother demanded the treatment, with the result that it was given by many physicians of various experiences and ability; consequently, much harm was done and not a few lives were lost.

The Journal of the A. M. A., (2) in an editorial, and again in answer to many inquiries, deplored the fact that a magazine of the standing of McClure's should have published such an article without first consulting some medical authority; but later showed by the (4) admission of J. W. Connally of the McClure Company, that the article was read and approved by both Professor Kroenig and Dr. Gauss before its publication.

The Henbane group from which Scopolamine is obtained is one of the oldest drugs known to the medical profession. It is highly probable that some preparation of this plant was the drug given the malefactors crucified by the Romans and refused by the Savior. It was known to the Arabians and used by them to render insensible the victims of their lust, and is also mentioned by Shakespeare as the drug by which Hamlet's father was poisoned. The principal action of the drug and its alkaloids is to produce a dreamy or forgetful delirium so that the patient has no recollection of events which take place during the period of its effect. The pain-relieving properties of opium are too well known to need comment.

In 1902 or 03, Scopolamine and Morphine were used in labor but abandoned on account of the number of asphyxiated children and other accidents that followed their administration. These accidents were probably due to an unstable preparation of the Scopolamine, for in the first one thousand cases reported from the Freiberg Clinic there was a large per cent. of asphyxiations; but as soon as a stable preparation was obtained this objection was removed and in the last four thousand cases reported the results have been better than without the use of these drugs.

Personally, I have always looked with favor on this treatment and when the first reports were published I tried it in a few cases but abandoned it on account of the excitement produced in one patient. There is no doubt that if a stable preparation of standard strength can be obtained and it is scientifically administered, at least 75 per cent of the cases can be carried successfully through labor without any, or with only a very slight recollection of what has occurred. In the majority of the remaining cases great benefit will be derived: the nervous strain will be relieved, and while there will be some recollection, the patient will be free from the fatigue which is usually felt for the next few days. In a small minority there will be no effect produced. Even where there is an idiosyncrasy the bad effects are not remembered by the patient.

The only objections that can be found are the nervousness and restlessness produced in a few individuals, and the bad effect sometimes produced on the respiration, but these can be avoided by carefully watching the initial dose.

It is impossible to formulate a definite rule in the administration of these drugs for the reasons mentioned above; but I have tried to follow that suggested by Professor Kroenig in his address before the Chicago Gynecological Society, except that I have not repeated the doses so often and have perhaps, waited a little longer to begin the treatment. In my twenty-one cases, I have been governed more by the severity and frequency of the pain than by the duration of labor. In two cases I have delayed the administration several hours because the pains were only slight and apparently doing no good. I believe, however, that labor would have been shortened had I used it sooner.

In nine of my cases there was complete amnesia for a period varying from one to three hours previous to the actual delivery. In one of these cases I purposely refrained from repeating the Scopolamine when the patient began to show signs of memory, as the pains, while apparently hard, were doing no good. I finally had to deliver with forceps, but the head was on the perineum and the delivery was easy.

In seven cases there was a soothing effect and more or less drowsiness. The patient rested well between each pain but had some recollection after labor of what had occurred. In these cases a small amount of chloroform was given during the passage of the head over the perineum. In the remaining five the effect was very slight, partly for want of time. In one case it was only about twenty minutes from the first dose to the delivery; while in another it was fifty minutes. In the other three the drug did not seem to have any effect except to slightly redden the face.

I have not observed any bad effect, and no great change in either the pulse or respiration. The greatest change in the pulse rate was in a patient of twenty, whose pulse was 84 when the first dose was given, and when I gave the third it was 105.

All have recovered their memory within twenty to fifty minutes after delivery. There was no excessive hemorrhage. Only two of the cases discharged any large clots, one of them immediately and one about an hour and a half later. But in this case I do not believe that the treatment could be responsible, as a patient I delivered the day before, who refused to take the treatment, had a larger hemorrhage about the same time after delivery; but neither of these cases could be classed as a serious post-partum hemorrhage. I have given a half c. c. of pituitrin in perhaps 75 per cent. of my cases after the head is well in the vagina and believe that it is of benefit at this stage.

Convalescence has been about the same as in cases which were not given the treatment. I do not believe in pushing a patient out of bed and have made no difference in my after treatment in these and other cases.

The babies, so far as I can observe, are not unpleasantly affectcd by the drugs. There was no more trouble with these babies than would be found in a series of cases without the use of Scopolamine and Morphine. Two babies were slightly cyanosed and required artificial respiration. The others cried more or less vigorously. Two, after crying for a few minutes, seemed to sleep for awhile but soon become all right. One baby died of what was apparently uremic poisoning five days after delivery and one at about two months of broncho-pneumonia, or perhaps congenital atelectasis. Dr. Clarke saw this baby and made the above diagnosis when it was nearly dead; it having had "absent treatment" by a Christian Scientist for some time.

These cases are not selected. I have refused to give the treatment to only one woman who was willing to take it, since I began, last October, and I had to deliver her with forceps.

The surroundings have very little to do with the treatment. Most of my cases, however, have been in the hospital where they are not disturbed by the family. The main essentials are a reasonably quiet room and a good nurse.

I have never given more than three doses of Scopolamine to any one patient, and in one I got complete amnesia with only two. The greatest amount given was 1-60 of a grain divided as follows: 1-150 gr. with the initial dose of Morphine; one hour later 1-200 gr. and 1 1-2 to 2 hours later another 1-200 gr. The longest time, with one exception, from the initial dose to delivery was five hours and twenty minutes. The average time in nine cases was three hours and thirty minutes.

The first stage of labor is markedly shortened as is shown by perhaps 75 per cent. of my cases, and in two worthy of special mention, the patients had been in labor; one twelve, the other twenty hours without more than one inch of dilatation when the first dose was given. In less than five hours the first was delivered and in the other there was complete dilatation, with the head in the vagina in three and one-half hours. Where the anaesthesia is at all complete the patient does not assist the pain, consequently, part of the force is lost in the second stage. A small dose of pitruitin is of benefit at this time and I have never seen any bad effect from the administration, but large or repeated doses are to be condemned.

I am well satisfied with the results I have had so far and expect to continue its use.

(1) S. G. & O., May, 1914.

(2) June 6, 1914, p. 1815.

(3) do page 1829.

(4) Jour. A. M. A., June 13, 1914, p. 1914.

324-5-6 Candler Building.

